# Supplementary material for: Comparing lagged impacts of mobility changes and environmental factors on COVID-19 waves in rural and urban India: a Bayesian spatiotemporal modelling study
Source: medRxiv. 2024 Jun 14:2024.06.12.24308871. Preprint. [Version 1] doi: 10.1101/2024.06.12.24308871 (PMC11213100; doi:10.1101/2024.06.12.24308871)
Supplement: 1 [file NIHPP2024.06.12.24308871v1-supplement-1.pdf]

medRxiv preprint doi: https://doi.org/10.1101/2024.06.12.24308871; this version posted June 14, 2024. The copyright holder for this preprint (which was not certified by peer review) is the author/funder, who has granted medRxiv a license to display the preprint in perpetuity.
It is made available under a CC-BY 4.0 International license .

Supporting information

Figure S1. Regions in India investigated by this study and the number and density of population at district level (administrative level II) in 2020. Areas shaded in grey are areas for which no data is available.

Figure S2. Five periods for travel network modularity analysis (A): 1) Pre-pandemic period (15 weeks) from November 10, 2019 to February 22, 2020; 2) First lockdown (6 weeks), from March 22 to May 2, 2020, that included strict travel restrictions, stay-at home orders and closure of many businesses; 3) Pre-

medRxiv preprint doi: https://doi.org/10.1101/2024.06.12.24308871; this version posted June 14, 2024. The copyright holder for this preprint (which was not certified by peer review) is the author/funder, who has granted medRxiv a license to display the preprint in perpetuity.
It is made available under a CC-BY 4.0 International license .

second lockdown period (8 weeks) from January 31 to March 27, 2021; 4) Second lockdown (6 weeks) for the Delta wave, from April 18 to May 29, 2021; 5) post-second lockdown period (8 weeks), from November 7 to December 31, 2021, after travel restrictions for COVID-19 had been lifted in India.

Fig S3. Relative changes of outbound travel from districts across India during the pandemic compared with average pre-pandemic levels during the 12 weeks from November 10, 2019, to February 22, 2020. (**A**) Reductions of outbound flows under the first lockdown during the 6-week period from March 22 to May 2, 2020. (**B**) Changes in outflow during the 8-week period from January 31 to March 27, 2021, before the second lockdown. (**C**) Reductions of outflows during the 6-week second lockdown from April 18 to May 29, 2021. (**D**) Changes in outflow during the 8-week period from November 7 to December 31, 2021. Sub-division maps at administrative level I (state) and II (district) were obtained from the GADM version 3.6 (https://gadm.org/). Regions in which outflow data are not available are those represented in green. Areas shaded in grey are areas for which no data is available.

Table S1. Summary Statistics for data used for wave 1 and Delta wave spatiotemporal models

SI Delta Wave

Table S2. Wave 2: Adequacy results for models with DLNMs and increasing complexity.

Table S3. Wave 2: Adequacy results for models (without DLNMs) using 2-week lag covariates with increasing complexity.

Table S4. Model hyperparameters using a range of prior distributions in best fit model 4.1 for Delta Wave

Figure S4. Relative intra-district mobility during the Delta wave in India, standardised by pre-pandemic mean baseline levels of mobility for the first eight weeks of 2020 (December 29, 2019 – February 22, 2020) for each district. The weeks in 2021 investigated are numbered in maps. Areas shaded in grey are areas for which no data is available.

Figure S5. Stringency Index of COVID-19 intervention policy implemented during the Delta wave in India. The weeks in 2021 investigated are numbered in maps. Areas shaded in grey are areas for which no data is available.

Figure S6. Mean temperature at 2m above the surface during the Delta wave in India. The weeks in 2021 investigated are numbered in maps. Areas shaded in grey are areas for which no data is available.

medRxiv preprint doi: https://doi.org/10.1101/2024.06.12.24308871; this version posted June 14, 2024. The copyright holder for this preprint
(which was not certified by peer review) is the author/funder, who has granted medRxiv a license to display the preprint in perpetuity.
It is made available under a CC-BY 4.0 International license .

Figure S7. Accumulated weekly precipitation (metres) during the Delta wave in India. The weeks in 2021 investigated are numbered in maps. Areas shaded in grey are areas for which no data is available.

Figure S8. Relative humidity during the Delta wave in India. The weeks in 2021 investigated are numbered in maps. Areas shaded in grey are areas for which no data is available.

Figure S9. Downward ultraviolet (UV) radiation (KJ/m2 per hour) during the Delta wave in India. The weeks in 2021 investigated are numbered in maps. Areas shaded in grey are areas for which no data is available.

Figure S10. Weekly Rt derived from COVID-19 cases reported during the Delta wave in India. The weeks in 2021 investigated are numbered in maps. Areas shaded in grey are areas for which no data is available.

Figure S11. Pairwise Pearson correlations between weekly means of variables at district level during the Delta wave in India, 2021. R0: basic reproduction number. Rt: instantaneous reproduction number. ln_R: log(Rt/R0). Cases_rate: new COVID-19 cases reported per 1000 people. Cases_accu_rate: cumulative cases per 1000 people reported since the first week of the wave. mean_intra: intra-district relative mobility. d2m: relative humidity. t2m: mean temperature of air (°C at 2m above the surface of land, sea or inland waters). tp: precipitation (metres). uv: downward ultraviolet radiation. Stringency: index of COVID-19 intervention stringency. Holiday: days of public holidays in a week. pop_sum: total population of each district. pop_density: population number per km2 of each district.

Figure S12. Kendall rank correlations between weekly means of variables at district level during the Delta wave in India, 2021. R0: basic reproduction number. Rt: instantaneous reproduction number. ln_R: log(Rt/R0). Cases_rate: new COVID-19 cases reported per 1000 people. Cases_accu_rate: cumulative cases per 1000 people reported since the first week of the wave. mean_intra: intra-district relative mobility. d2m: relative humidity. t2m: mean temperature of air (°C at 2m above the surface of land, sea or inland waters). tp: precipitation (metres). uv: downward ultraviolet radiation. Stringency: index of COVID-19 intervention stringency. Holiday: days of public holidays in a week. pop_sum: total population of each district. pop_density: population number per km2 of each district.

Figure S13. Posterior predictive mean Rt during the Delta wave in India, 2021, derived from the best fitting model (model 4.1) at country level using leave-one-week-out cross-validation approach. The weeks in 2021 investigated are numbered in maps. Areas shaded in grey are areas for which no data is available.

medRxiv preprint doi: https://doi.org/10.1101/2024.06.12.24308871; this version posted June 14, 2024. The copyright holder for this preprint (which was not certified by peer review) is the author/funder, who has granted medRxiv a license to display the preprint in perpetuity.
It is made available under a CC-BY 4.0 International license .

Figure S14. Standard deviation (SD) of posterior predictive Rt during the Delta wave in India, 2021, derived from the best fitting model (model 4.1 without DLNMs) at country level using a leave-one-week-out cross-validation approach. Areas shaded in grey are areas for which no data is available.

Figure S15. Posterior predictive mean Rt during the Delta wave in India, 2021, derived from the best fitting model (model 4.1) at country level using leave-one-state-out cross-validation approach. The weeks in 2021 investigated are numbered in maps. Areas shaded in grey are areas for which no data is available.

Figure S16. Standard deviation (SD) of posterior predictive Rt during the Delta wave in India, 2021, derived from the best fitting model (model 4.1 without DLNMs) at country level using a leave-one-state-out cross-validation approach. Areas shaded in grey are areas for which no data is available.

Figure S17. Contribution of spatial random effects to estimates of Rt changes in the base model. Areas shaded in grey are areas for which no data is available.

Figure S18. Improvement by using the best fitting model across the country, compared to baseline model. Difference between mean absolute error (MAE) for the baseline model (weekly random effects, spatial random effects and population density) and MAE for the best fitting model (model 4.1 with DLNMs). Districts with positive values (pink) suggest that capturing the nonlinear and delayed impacts of mobility, climate information and intervention stringency, improves the model in these areas. Districts with negative values (blue) suggest that mobility, intervention and climate information did not improve the model fit and other unexplained factors might dominate space-time dynamics in these areas. The MAE of the selected model was smaller than the baseline model for 385 of the 665 (57.9%) districts in India, with the results of model performance provided by geo-political regions in the Table. Areas shaded in grey are areas for which no data is available.

Figure S19. Observed versus posterior fitted Rt in the capital district of each state using the best fitting model (model 4.1 with DLNMs) at country level. Graphs with a log scale at y-axis show the observed Rt derived from reported case data, and corresponding mean and 95% confidence interval (CI, shaded pink area) of fitted Rt, derived from the best fitting model (model 4.1 with DLNMs) at country level. States are ordered by their geographical location.

Figure S20. Observed versus posterior predictive Rt in the capital district of each state, using leave-one-week-out cross-validation approach**.** Graphs with a log scale at y-axis show the observed Rt derived from reported case data, and corresponding posterior predictive mean and 95% prediction interval (CI, shaded pink area), derived from the best fitting model (model 4.1 with DLNMs) at country level. States are ordered by their geographical location.

medRxiv preprint doi: https://doi.org/10.1101/2024.06.12.24308871; this version posted June 14, 2024. The copyright holder for this preprint (which was not certified by peer review) is the author/funder, who has granted medRxiv a license to display the preprint in perpetuity.
It is made available under a CC-BY 4.0 International license .

Figure S21. Contribution of spatial random effects to estimates of Rt changes in the base model. Areas shaded in grey are areas for which no data is available.

Figure S22. Improvement of using the best fitting model with 2-week lag covariates (no DLNMs), compared to baseline model with the same lag. Difference between mean absolute error (MAE) for the baseline model and MAE for the best fitting model (Model 4.1). Districts with positive values (pink) suggest that capturing the 2-week lag impacts of mobility, temperature, UV and intervention stringency, improves the model in these areas. Districts with negative values (blue) suggest that mobility, intervention and climate information did not improve the model fit and other unexplained factors might dominate space-time dynamics in these areas. The MAE of the selected model was smaller than the baseline model for 428 of the 665 (64.4%) districts in India, and further improved the best fitting model with DLNMs (Figure S12). Results of model performance are provided by geo-political regions in the Table. Areas shaded in grey are areas for which no data is available.

Figure S23. Posterior predictive mean Rt during the Delta wave in India, 2021, derived from the best fitting model (model 4.1 without DLNMs) at country level using 2-week lag covariates and leave-one-week-out cross-validation approach. Areas shaded in grey are areas for which no data is available.

Figure S24. Standard deviation (SD) of posterior predictive Rt during the Delta wave in India, 2021, derived from the best fitting model (model 4.1 without DLNMs) at country level using 2-week lag covariates and leave-one-week-out cross-validation approach. Areas shaded in grey are areas for which no data is available.

Fig S25. Observed versus posterior predictive Rt in the capital district of each state. Graphs with a log scale at y-axis show the observed Rt derived from reported case data, and corresponding posterior predictive mean and 95% prediction interval (CI, shaded pink area), derived from the best fitting model without DLNMs at country level (model 4.1: base model + mobility + temperature + UV + intervention policy; see SI Table S2), using 2-week lag covariates and leave-one-week-out cross-validation approach. States are ordered by their geographical location.

Figure S26. Posterior predictive mean Rt during the Delta wave in India, 2021, derived from the best fitting model (model 4.1 without DLNMs) at country level using 2-week lag covariates and leave-one-state-out cross-validation approach. Areas shaded in grey are areas for which no data is available.

Figure S27. Standard deviation (SD) of posterior predictive Rt during the Delta wave in India, 2021, derived from the best fitting model (model 4.1 without DLNMs) at country level using 2-week lag

medRxiv preprint doi: https://doi.org/10.1101/2024.06.12.24308871; this version posted June 14, 2024. The copyright holder for this preprint (which was not certified by peer review) is the author/funder, who has granted medRxiv a license to display the preprint in perpetuity.
It is made available under a CC-BY 4.0 International license .

covariates and leave-one-state-out cross-validation approach. Areas shaded in grey are areas for which no data is available.

SI Wave 1

Table S5. Wave 1: Adequacy results for models with DLNMs and increasing complexity.

Table S6. Wave 1: Adequacy results for models (without DLNMs) using 2-week lag covariates with increasing complexity.

Table S7. Model hyperparameters using a range of prior distributions in best fit model 4.1 for Wave 1

Figure S28. COVID-19 cases reported by district each week during wave 1 in India. The weeks in 2020 investigated are numbered in maps. Areas shaded in grey are areas for which no data is available.

Figure S29. Relative intra-district mobility during wave 1 in India, standardised by pre-pandemic mean baseline levels of mobility for the first eight weeks of 2020 (December 29, 2019 – February 22, 2020) for each district. The weeks in 2020 investigated are numbered in maps. Areas shaded in grey are areas for which no data is available.

Figure S30. Stringency Index of COVID-19 intervention policy implemented during wave 1 in India. The weeks in 2020 investigated are numbered in maps. Areas shaded in grey are areas for which no data is available.

Figure S31. Mean temperature at 2m above the surface during wave 1 in India. The weeks in 2020 investigated are numbered in maps. Areas shaded in grey are areas for which no data is available.

Figure S32. Accumulated weekly precipitation (metres) during wave 1 in India. The weeks in 2020 investigated are numbered in maps. Areas shaded in grey are areas for which no data is available.

Figure S33. Relative humidity during wave 1 in India. The weeks in 2020 investigated are numbered in maps. Areas shaded in grey are areas for which no data is available.

Figure S34. Downward ultraviolet (UV) radiation (KJ/m2 per hour) during wave 1 in India. The weeks in 2020 investigated are numbered in maps. Areas shaded in grey are areas for which no data is available.

medRxiv preprint doi: https://doi.org/10.1101/2024.06.12.24308871; this version posted June 14, 2024. The copyright holder for this preprint (which was not certified by peer review) is the author/funder, who has granted medRxiv a license to display the preprint in perpetuity.
It is made available under a CC-BY 4.0 International license .

Figure S35. Weekly Rt derived from COVID-19 cases reported during the wave 1 in India. The weeks in 2020 investigated are numbered in maps. Areas shaded in grey are areas for which no data is available.

Figure S36. Pairwise Pearson correlations between weekly means of variables at district level during the wave 1 in India, 2020. R0: basic reproduction number. Rt: instantaneous reproduction number. ln_R: log(Rt/R0). Cases_rate: new COVID-19 cases reported per 1000 people. Cases_accu_rate: cumulative cases per 1000 people reported since the first week of the wave. mean_intra: intra-district relative mobility. d2m: relative humidity. t2m: mean temperature of air (°C at 2m above the surface of land, sea or inland waters). tp: precipitation (metres). uv: downward ultraviolet radiation. Stringency: index of COVID-19 intervention stringency. Holiday: days of public holidays in a week. pop_sum: total population of each district. pop_density: population number per km2 of each district.

Figure S37. Kendall rank correlations between weekly means of variables at district level during the wave 1 in India, 2020. R0: basic reproduction number. Rt: instantaneous reproduction number. ln_R: log(Rt/R0). Cases_rate: new COVID-19 cases reported per 1000 people. Cases_accu_rate: cumulative cases per 1000 people reported since the first week of the wave. mean_intra: intra-district relative mobility. d2m: relative humidity. t2m: mean temperature of air (°C at 2m above the surface of land, sea or inland waters). tp: precipitation (metres). uv: downward ultraviolet radiation. Stringency: index of COVID-19 intervention stringency. Holiday: days of public holidays in a week. pop_sum: total population of each district. pop_density: population number per km2 of each district.

Figure S38. Posterior predictive mean Rt during wave 1 in India, 2020, derived from the best fitting model (model 4.1) at country level using leave-one-week-out cross-validation approach. The weeks in 2020 investigated are numbered in maps. Areas shaded in grey are areas for which no data is available.

Fig S39. Standard deviation (SD) of posterior predictive Rt during wave 1 in India, 2020, derived from the best fitting model (model 4.1) at country level leave-one-week-out cross-validation approach. Areas shaded in grey are areas for which no data is available.

Figure S40. Posterior predictive mean Rt during wave 1 in India, 2020, derived from the best fitting model (model 4.1) at country level using leave-one-district-out cross-validation approach. The weeks in 2020 investigated are numbered in maps. Areas shaded in grey are areas for which no data is available.

medRxiv preprint doi: https://doi.org/10.1101/2024.06.12.24308871; this version posted June 14, 2024. The copyright holder for this preprint (which was not certified by peer review) is the author/funder, who has granted medRxiv a license to display the preprint in perpetuity.
It is made available under a CC-BY 4.0 International license .

Fig S41. Standard deviation (SD) of posterior predictive Rt during wave 1 in India, 2020, derived from the best fitting model (model 4.1) at country level leave-one-district-out cross-validation approach. Areas shaded in grey are areas for which no data is available.

Figure S42. Contribution of spatial random effects to estimates of Rt changes in the base model. Areas shaded in grey are areas for which no data is available.

Figure S43. Improvement by using the best fitting model across the country, compared to baseline model. Difference between mean absolute error (MAE) for the baseline model (weekly random effects, spatial random effects and population density) and MAE for the best fitting model (model 4.1 with DLNMs). Districts with positive values (pink) suggest that capturing the nonlinear and delayed impacts of mobility, climate information and intervention stringency, improves the model in these areas. Districts with negative values (blue) suggest that mobility, intervention and climate information did not improve the model fit and other unexplained factors might dominate space-time dynamics in these areas. The MAE of the selected model was smaller than the baseline model for 430 of the 661 (65.17%) districts in India, with the results of model performance provided by geo-political regions in the Table. Areas shaded in grey are areas for which no data is available.

Figure S44. Observed versus posterior fitted Rt in the capital district of each state using the best fitting model (model 4.1 with DLNMs) at country level. Graphs with a log scale at y-axis show the observed Rt derived from reported case data, and corresponding mean and 95% confidence interval (CI, shaded pink area) of fitted Rt, derived from the best fitting model (model 4.1 with DLNMs) at country level. States are ordered by their geographical location.

Figure S45. Observed versus posterior predictive Rt in the capital district of each state, using leave-one-week-out cross-validation approach**.** Graphs with a log scale at y-axis show the observed Rt derived from reported case data, and corresponding posterior predictive mean and 95% prediction interval (CI, shaded pink area), derived from the best fitting model (model 4.1 with DLNMs) at country level. States are ordered by their geographical location.

Figure S46. Posterior predictive mean Rt during the wave 1 in India, 2020, derived from the best fitting model (model 4.1 without DLNMs) at country level using 2-week lag covariates and leave-one-week-out cross-validation approach. Areas shaded in grey are areas for which no data is available.

Fig S47. Standard deviation (SD) of posterior predictive Rt during wave 1 in India, 2020, derived from the best fitting model (model 4.1 without DLNMs) at country level using 2-week lag covariates and

medRxiv preprint doi: https://doi.org/10.1101/2024.06.12.24308871; this version posted June 14, 2024. The copyright holder for this preprint (which was not certified by peer review) is the author/funder, who has granted medRxiv a license to display the preprint in perpetuity.
It is made available under a CC-BY 4.0 International license .

leave-one-week-out cross-validation approach. Areas shaded in grey are areas for which no data is available.

Figure S48. Posterior predictive mean Rt during the wave 1 in India, 2020, derived from the best fitting model (model 4.1 without DLNMs) at country level using 2-week lag covariates and leave-one-district-out cross-validation approach. Areas shaded in grey are areas for which no data is available.

Fig S49. Standard deviation (SD) of posterior predictive Rt during wave 1 in India, 2020, derived from the best fitting model (model 4.1 without DLNMs) at country level using 2-week lag covariates and leave-one-district-out cross-validation approach. Areas shaded in grey are areas for which no data is available.
